# Impact of flavorants on nicotine vapor self-administration in adolescent mice: Cherry on top?

**DOI:** 10.1016/j.jpet.2025.103700

**Published:** 2025-09-09

**Authors:** Kendra M. Loedige, Jibran Y. Khokhar

**Affiliations:** Department of Anatomy and Cell Biology, Schulich School of Medicine and Dentistry, Western University, London, Ontario, Canada

**Keywords:** Vaping, Flavors, Vanilla, Cherry

Over the past decade, the rapid rise in popularity of electronic nicotine delivery systems (ENDS) has made these products the most commonly used source of nicotine among US adolescents, resulting in a substantial increase in youth nicotine exposure during this critical developmental period.^1^, ^2^, ^3^ Initially developed as harm reduction tools for adults seeking alternatives to combustible cigarettes, ENDS products now contain a diverse range of palatable e-liquid flavors, mimicking desserts, fruit, or candy, and have been increasingly marketed toward and adopted by youth.^4^^,^^5^ Observational studies suggest that among adolescents, flavorants increase willingness to initiate use,^6^ reduce perceived health risks,^7^^,^^8^ and contribute to sustained use.^9^ However, controlled studies are needed to determine whether flavorants directly drive ENDS uptake and abuse liability. While regulatory efforts have largely focused on nicotine content, growing preclinical evidence indicates that certain flavorants can directly engage reward pathways, potentially contributing to dependence.^10^^,^^11^

In a recent issue of JPET, Swenson et al^12^ investigated whether common flavorants support reinforcement independently or in combination with nicotine in an adolescent mouse model using the e-Vape self-administration (EVSA) paradigm.^12^ Male and female adolescent mice self-administered vaporized propylene glycol/vegetable glycerin vehicle with vanilla (vanillin and ethyl vanillin) or cherry (ethyl vanillin, vanillin, ethyl acetate, ethyl maltol, and maltol) flavorants, with or without nicotine, at commercially relevant concentrations. Importantly, vanilla flavorant alone supported robust self-administration in both nicotine-naïve mice and mice pre-exposed to nicotine with menthol. This was demonstrated by increased active nose pokes, escalated responding, and progressive ratio breakpoints comparable to those induced by nicotine, suggesting that vanilla can act as a primary reinforcer. By contrast, the cherry flavorant delivered through EVSA did not support behavioral reinforcement.

Using fast-scan cyclic voltammetry, passive exposure to vanilla vapor was found to significantly elevate both tonic and phasic dopamine release in the nucleus accumbens, a central node in the mesolimbic reward system activated by nicotine and other drugs of abuse. These transients, signaling reward salience, suggest that vanilla flavorants robustly engage dopaminergic circuits. While fast-scan cyclic voltammetry offers high temporal resolution, especially in in vitro preparations, fiber photometry using genetically encoded dopamine sensors can link in vivo dopamine release to discrete events, such as cues and vapor delivery, across extended behavioral sessions.^13^ Additionally, fiber photometry allows for the simultaneous capture of both slow and phasic dopamine changes within an in vivo session, as well as the potential to genetically target distinct cellular compartments, providing the ability to create “synaptic” dopamine release.^13^ Using this approach, Swenson et al^12^ found that vanilla flavorant alone was able to elicit dopamine transients in response to the cue predictive of vapor delivery and vapor delivery itself, which were comparable in magnitude to those induced by nicotine.

At the molecular level, docking simulations suggest that the primary constituents of the vanilla flavorant, vanillin and ethyl vanillin, bind to the *α*4*β*2 nicotinic acetylcholine receptor (nAChR), a subtype central to nicotine’s reinforcing effects. This receptor can assemble into 2 stoichiometries with differing sensitivities to acetylcholine: high- (*α*4_[2]_*β*2_[3]_) and low-sensitivity (*α*4_[3]_*β*2_[2]_).^14^ Interestingly, computational modeling predicts that vanilla flavorants interact with residues similar to those bound to green apple flavorants, which have been shown to stabilize the low-sensitivity form.^10^^,^^11^ In contrast, nicotine preferentially upregulates the high-sensitivity form.^15^ These docking-based predictions, while not yet functionally validated, suggest that vanilla flavorants may exert pharmacological effects through direct receptor engagement in a manner mechanistically distinct from nicotine, potentially contributing to their reinforcing properties in the absence of nicotine.

A key limitation of EVSA paradigms is that they inherently engage orosensory pathways, making it challenging to disentangle the pharmacological effects of flavorants from their sensory properties. The conditioned place preference (CPP) paradigm could address this limitation by isolating the effects of flavorants delivered systemically, bypassing oral and nasal sensory stimuli.^16^ Using CPP, intraperitoneal delivery of menthol has been shown to enhance nicotine-induced CPP,^17^ while green apple flavorants elicit CPP even in the absence of nicotine, supporting their ability to reinforce behavior directly through their action on nAChRs.^18^ Given prior exposure to menthol in one cohort of animals, it would be important to assess whether prior orosensory engagement through exposure to other flavorants could modify the reinforcing properties of subsequent flavorants.

In addition to their intrinsic pharmacological effects, further work is needed to investigate potential pharmacokinetic interactions between vanilla flavorants and nicotine. Menthol has been shown to suppress respiration and increase nicotine retention in the lungs,^19^ and cherry-flavored e-liquids have been shown to produce greater nicotine delivery compared with vanilla.^20^ Nicotine is primarily metabolized by the hepatic enzyme CYP2A6, which accounts for approximately 80% of its clearance and exhibits significant genetic variability.^21^ Several e-liquid flavorants, including cinnamaldehyde, benzaldehyde, vanillin, and ethyl vanillin, have been shown to inhibit CYP2A6 activity in vitro, potentially prolonging nicotine’s bioavailability.^22^ Ethyl vanillin, when administered systemically, increases nicotine concentrations in rat blood and demonstrates greater binding affinity to CYP2A6 than nicotine itself, suggesting direct enzymatic competition.^23^ These findings raise important questions about the effects of vaporized delivery and chronic exposure to vanilla flavorants on nicotine pharmacokinetics, especially in individuals with genetically reduced CYP2A6 activity. Furthermore, because most commercial e-liquids contain variable mixtures of delivery solvents, multiple flavorants, and other constituents (eg, sweeteners and synthetic coolants), future research should investigate potential additive, synergistic, or antagonistic interactions among these components, and how combinations, when heated and aerosolized, affect nicotine absorption, metabolism, and dependence liability.^24^^,^^25^

Developmental stage may also modulate the reinforcing effects of flavorants with and without nicotine, with adolescence representing a unique window of vulnerability.^26^ Adolescents tend to exhibit heightened sensitivity to rewarding stimuli and diminished sensitivity to aversive outcomes, which may place them at increased risk of nicotine dependence.^27^ Adolescent rats have shown greater nicotine-induced CPP compared with adults, suggesting enhanced sensitivity to its rewarding effects.^28^ Adolescent female rats have also been shown to display a greater approach behavior toward nicotine vapor ports than both adolescent males and adults, highlighting age-by-sex (biological classification as male or female) interactions in vapor reinforcement.^29^ These findings align with broader evidence that adolescence is characterized by increased risk-taking and novelty-seeking, potentially amplifying the vulnerability and reinforcing properties of nicotine and vaping-related cues. Interestingly, in passive vapor exposure, adult females have also been shown to exhibit the greatest nicotine-induced CPP, elevated nicotine and cotinine levels in blood and brain tissue, and reductions in functional connectivity in regions associated with nicotine dependence and contextual cue reactivity, greater than in adolescent or male counterparts.^30^ These data highlight that susceptibility to nicotine reinforcement is shaped by both age and sex, further adding complexity to the interactions between nicotine and flavorants.

Age also influences the ability to form conditioned flavor preferences, which may, in turn, affect the reinforcing drive of e-liquid flavorants. In flavor preference tasks, younger rats form stronger and longer-lasting flavor-reward associations than their older counterparts. For instance, rats trained at 7 months to associate cherry or grape flavors with sucrose in solution later preferred the previously sweetened flavor when tested with unsweetened solutions, whereas 24-month-old rats did not exhibit a preference.^31^ Similarly, flavor preferences acquired in 3-week-old rats persisted into adulthood, demonstrating the lasting effect of flavor-reward associations.^32^ Taste sensitivity and preferred concentration for flavorants also shift with age,^33^ which may modulate the salience and reinforcing value of e-liquid flavorants across developmental stages. These findings reinforce that adolescence is not only a period of increased susceptibility to nicotine’s pharmacological effects, but also a critical window for learning and reinforcing flavor-based cues, further compounding the risks associated with flavored ENDS.

Given the heightened capacity of adolescents to form persistent flavor-reward associations, it is important to consider how prior exposure to flavorant analogs outside the ENDS context may influence their reinforcing value. In the EVSA paradigm described by Swenson et al,^12^ rodents first encountered flavorants during vapor self-administration. In contrast, human adolescents often encounter similar flavorants in foods (eg, vanilla ice cream) associated with reward (eg, sugar), where they may become conditioned reinforcers prior to nicotine exposure.^34^ Such pre-existing associations could enhance the salience and reinforcing value of flavored ENDS products.^34^ To improve the translational validity of EVSA paradigms to understand flavorant reinforcement and disentangle the contribution of learned flavor-reward associations, future studies could incorporate preconditioning paradigms in which flavorants are first paired with sucrose to establish them as conditioned stimuli prior to EVSA testing. This approach might provide a more realistic assessment of how learned flavor-reward associations influence vaping behavior and escalation.

Finally, exposure to nicotine and flavorants during adolescence may have lasting effects on behavior and neurobiology. Adolescent rats have been shown to display reduced sensitivity to nicotine withdrawal compared with adults, which is thought to amplify the reinforcing effects of nicotine by minimizing its aversive consequences.^35^ It is worth exploring whether this could be amplified by flavorants, which may mask negative physiological cues or further suppress aversive withdrawal symptoms, increasing continued use and dependence. Additionally, adolescent male rats exposed to vaporized nicotine displayed enhanced sign-tracking behavior, a proxy for incentive salience attribution in adulthood.^36^ Given that flavorants have been shown to independently engage reward pathways, further work should consider the impact of adolescent exposure to vaporized flavorants on reward-seeking behavior later in life.

Together, these findings challenge the assumption that flavored nicotine-free ENDS are without abuse liability. Swenson et al^12^ demonstrated that flavorants, such as vanilla, can directly engage reward circuits, elevate dopamine release, and support reinforcement in the absence of nicotine.^12^ Regulatory frameworks must broaden their focus beyond nicotine content to account for the pharmacological and behavioral effects of flavorants. Restricting access to flavored ENDS products may be a crucial step toward curbing early experimentation and dependence trajectories. Ultimately, this work underscores that flavorants are not passive additives, but active agents capable of shaping the course of addiction. Ongoing research is needed to refine our understanding of their mechanisms, developmental impacts, and implications for public health, where they are most likely to drive problematic use.

## Conflict of interest

The author declares no conflicts of interest.
